# Serum p-Cresol and 7-HOCA Levels and Fatty Acid and Purine Metabolism Are Associated with Survival, Progression, and Molecular Classification in GB—Serum Proteome and Metabolome Analysis Pre vs. Post Up-Front Chemoirradiation

**DOI:** 10.3390/curroncol32110650

**Published:** 2025-11-20

**Authors:** Andra V. Krauze, M. Li, Y. Zhao, E. Tasci, S. Chappidi, T. Cooley Zgela, M. Sproull, M. Mackey, K. Camphausen

**Affiliations:** 1 Radiation Oncology Branch, Center for Cancer Research, National Cancer Institute, NIH, 9000 Rockville Pike, Building 10, CRC, Bethesda, MD 20892, USA; erdal.tasci@nih.gov (E.T.); shreya.chappidi@nih.gov (S.C.); theresa.cooleyzgela@nih.gov (T.C.Z.); sproullm@mail.nih.gov (M.S.); mmackey@mail.nih.gov (M.M.); camphauk@mail.nih.gov (K.C.); 2 Computational and Systems Biology Branch, Biometric Research Program, Division of Cancer Treatment and Diagnosis, National Cancer Institute, NIH, 9000 Rockville Pike, Building 10, CRC, Bethesda, MD 20892, USA; limingc@mail.nih.gov (M.L.); zhaoy@mail.nih.gov (Y.Z.); 3 Department of Computer Science and Technology, University of Cambridge, 15 JJ Thomson Ave, Cambridge CB3 0FD, UK

**Keywords:** glioma, survival, progression, molecular classification, proteome, metabolome

## Abstract

Background: Glioblastoma (GB) is the most common primary brain tumor, with poor prognosis, significant neurological symptoms, and near-universal recurrence. Biomarker development is often limited by the scarcity of tumor tissue available for study. Noninvasive serum-based profiling offers potential to improve outcomes. Purpose: This study examined serum proteomic and metabolomic profiles pre- and post-concurrent chemoirradiation (CRT) to identify associations with patient outcomes and molecular classification, and to explore relevant signaling and metabolic pathways. Methods: Serum samples from 109 GB patients, obtained prior to and following completion of CRT, were analyzed with each patient serving as their own control, using a SOMAScan^®^ proteomic assay (7289 proteins) and metabolomics (SECIM, 6015 compounds). Clinical data were obtained through chart review. Proteomic and metabolomic changes were examined at baseline (prior to CRT) and in alteration (pre- vs. post-CRT) for their association with overall survival (OS), progression-free survival (PFS), MGMT, and IDH status. Cox models, gene set enrichment analysis (Hallmark, GSEA), and Kaplan–Meier survival analysis were used. Results: Several hundred proteins and metabolites were associated with OS and PFS. MGMT status was known in 60% and IDH in 38% of patients. Pre-CRT DLST (HR 11.7, *p* < 0.001, adj *p* = 0.01) was the only protein significantly associated with OS. Pre-CRT, and higher 7-HOCA was linked to worse OS (HR 1.3) and PFS (HR 1.5), while increased p-cresol was associated with improved OS (HR 0.8) and PFS (HR 0.9). Kaplan–Meier analysis based on signal alteration post-CRT vs. pre-CRT, revealed superior OS with lower DLST and MSR1 and superior PFS with higher PGAM2 and ATG5, and lower 7-HOCA. Pathway analysis linked improved PFS to fatty acid metabolism, citric acid cycle, and purine biosynthesis. MGMT and IDH class comparisons revealed associations primarily with amino acid and fatty acid metabolism. Both MGMT methylation and IDH mutation correlated with increased PLAG12B expression, with significance only for MGMT (*p* < 0.001). IDH mutation was associated with decreased MSR1 (*p* = 0.047) and p-cresol (*p* < 0.001). Conclusions: Serum-based fatty acid and purine metabolism pathways are associated with OS and PFS in GB. 7-HOCA and p-cresol emerged as potential biomarkers linked to treatment response and molecular subtype. These findings support further investigation of noninvasive biospecimens for clinically actionable biomarkers in GB.

## Simple Summary

Glioblastoma is the most common and aggressive brain cancer. Even with surgery, chemotherapy, and radiation, it almost always returns because the tumor becomes resistant to treatment. Doctors currently lack simple blood tests that can show how a patient’s tumor is responding to better guide therapy choices. In this study, we analyzed blood samples from people with glioblastoma taken before and after treatment. We measured thousands of proteins and small molecules and identified several linked to how long patients lived and to when their cancer progressed. Two molecules in particular, p-cresol and 7-HOCA, stood out as strong indicators of outcome. Changes in how the body uses energy, fats, and building blocks of DNA also reflected tumor behavior. These findings show that blood can carry important signals about a brain tumor’s biology and may one day help doctors monitor treatment and design more personalized therapies.

## 1. Introduction

Glioblastoma (GB) is the most common and the most aggressive brain tumor [1]. The current standard of care involves maximal surgical resection followed by concurrent radiation therapy (RT) and temozolomide (TMZ), followed by adjuvant TMZ [2]. There is currently a lack of clinical biomarkers in glioma that can be detected in minimally invasive biospecimens and are directly linked to progression or survival, with realistic prospects for implementation into clinical use [3]. While MGMT methylation status is the single prognostic and predictive marker with MGMT-methylated patients having superior response to systemic management, improved progression, and overall survival [4], and IDH mutation status has redefined GB classification with IDH-mutated tumors now reclassified given superior outcome [5], both molecular features are detected in tumor tissue. This analysis can require several weeks to return results, which do not provide a distinct path towards harnessing tumor signaling with targeted agents or RT optimization. The exact mechanisms by which they exert their impact on the outcome are not fully understood. The paucity of data and, when available, the pervasive class imbalance in MGMT and IDH status annotation in public datasets pose a barrier to advancement in this direction for all biospecimens [6,7,8]. From an imaging perspective, while patients are currently followed with MRI of the brain every 2–3 months following completion of up front CRT, MRI imaging of the brain remains difficult to interpret posttreatment indicating possible pseudoresponse or pseudoprogression and there is currently no biomarker that predicts for early progression and allows for early intervention or selection of patients for personalized management given evolving novel agents [9,10,11]. The use of MRI as a data stream is limited by the complexity of the data and the restrictions on data sharing. The challenge with evolving biomarkers has been reproducibility across studies, matching biomarkers to outcomes as well as glioma subtypes, and correlation of markers across different biospecimens. Given the cost and practicality of acquisition, as well as the ability to rapidly grow large-scale data for computational analysis, blood is the most intuitive avenue as the noninvasive biospecimen of choice. Glioma patients already undergo weekly blood work to monitor temozolomide side effects as part of up-front management, providing an intuitive means of leveraging blood as the preferred biospecimen for biomarker development. The analysis of noninvasive biospecimen provides a compelling rationale for the advancement of both biological understanding of the disease and resistance to treatment as well as path to personalization of management given critical tumor heterogeneity [12]. A number of studies are increasingly employing multichannel data to identify pivotal pathways [13,14,15,16,17]; however, most studies are currently based on tumor tissue acquired at a single timepoint [18,19]. The present study involves serum as the biospecimen analyzed, obtained prior to and following completion of up-front CRT. A subset of patients also received valproic acid during the trial, and their analysis has been reported elsewhere [20,21]. In this study, we aimed to examine how differential alteration in proteomic and metabolomic expression pre- vs. post-completion of concurrent chemoirradiation (CRT) is associated with OS and PFS and molecular classification in GB.

## 2. Methods

### 2.1. Patients

A total of 109 patients were included in the analysis (Supplementary Table S1). One hundred and nine had specimens for proteomic analysis, and of these, 107 had sufficient specimens for metabolomic analysis. Twenty-nine patients had received concurrent valproic acid (VPA) and had been subjected to class comparison analysis elsewhere [20]. All patients had pathology-proven GB (diagnosed between 2005 and 2013) and were enrolled in NCI- and NIH-IRB-approved protocols. For each individual patient of the 109 total patients, blood biospecimens were obtained before and after CRT completion. All samples were included in the study, with acquisition, storage, and annotation previously described [22]. Serum samples were screened using the multiplexed, aptamer-based approach (SOMAScan^®^ assay) to measure the relative concentrations of 7596 protein targets (7289 human) for changes in expression using approximately 150 μL of serum [23,24]. Clinical data (age and sex), tumor characteristics (location and MGMT methylation status), management-related factors (extent of resection), radiation therapy volumes (GTV T1), recursive partitioning analysis score (RPA) [25], and outcomes (progression-free survival (PFS), overall survival (OS)) were obtained or derived (RPA) from the protocol database and electronic health record with GTV T1 generated per ICRU report 83 [26] obtained from the radiation therapy treatment planning (contoured on the T1 Gadolinium sequence of the MRI scan employed for RT planning per standard guidelines).

### 2.2. Survival Analysis

Univariate Cox proportional hazards models were fit to test individual protein or metabolite expression pre-treatment or change levels after treatment for association with both OS and PFS. Regression coefficients from these models were tested using a two-sided Wald test, considering *p*-values < 0.05 as significant. The association between the expression of individual proteins or metabolites and survival was examined, adjusted for age, GTV T1, and VPA. Kaplan–Meier survival curves were plotted using the R statistical package 4.3.0.

### 2.3. Class Comparisons

Tests for differences in clinical characteristics and protein expression changes between the MGMT-methylated and unmethylated classes, and the IDH-mutated and wild-type classes, were conducted using two-sided *t*-tests, with *p*-values of <0.05 considered significant. Adjustments for age, sex, and other clinical covariates were made separately when appropriate. The Benjamini and Hochberg method was used to estimate the false discovery rate [27].

### 2.4. Gene Set Enrichment Analysis

In survival analysis of proteomic data, GSEA was performed on a curated set of 98 metabolic pathways previously defined in a study by Gaude and Frezza [28]. We used the resulting test statistics as input for a pathway enrichment analysis using a software package developed in-house (available upon request). The KS statistic was used to determine the significance of pathway enrichment. The analysis for over-represented metabolites was carried out using the publicly available tool Metaboanalyst, Metabolite Set Enrichment Analysis (MSEA), to determine if metabolites were enriched and linked to mechanistic processes [29], in addition to using 3694 metabolite and lipid pathways from RaMP-DB (integrating KEGG via HMDB, Reactome, and WikiPathways). In MGMT and IDH status comparison, Gene Set Enrichment Analyses (GSEA) were performed in BRB-ArrayTools [30] on 50 Cancer Hallmark gene sets downloaded from MSigDB. The Kolmogorov–Smirnov (KS) tests are applied separately to each of the 50 gene sets. A gene set is considered significant if its corresponding KS re-sampling *p*-value is below the specified threshold (*p*-values < 0.05).

## 3. Results

### 3.1. Survival Analysis and Differential Protein and Metabolite Expression

One hundred and nine patients were included in the proteomic analysis, and 107 had sufficient samples for metabolomic analysis (Supplementary Table S1). Adjustment for age, VPA, and GTV T1 was performed due to their significant impact on outcome (Supplementary Figure S1). Several hundred proteins were identified in association with OS, PFS, MGMT, and IDH class comparisons (Supplementary Figure S2).

### 3.2. Proteomic and Metabolomic Data Analyses

All 7289 human proteins present in the 7K Somalogic panel were included in the analysis, with each protein uniquely identified by its sequence ID. In order to determine relationships between protein expression and patient outcomes, individual protein expression pre-treatment or change levels after treatment were tested for association with both OS and PFS. Proteins associated with survival and progression are shown in Supplementary Figure S2. Several hundred proteins were identified in association with OS, PFS, and MGMT and IDH class comparisons (Supplementary Figure S2). Overall, only one protein met significance for OS after adjustment for multiple comparisons (FDR *p* < 0.05), dihydrolipoamide S-succinyltransferase (DLST), with an increase in DLST level pre-CRT associated with a significant detriment in survival (HR 11.7, *p* < 0.001, *p* adj = 0.01). Several other proteins emerged in the analysis, including for PFS; however, the adjusted *p*-values were higher (Table 1).

**Figure 1:**
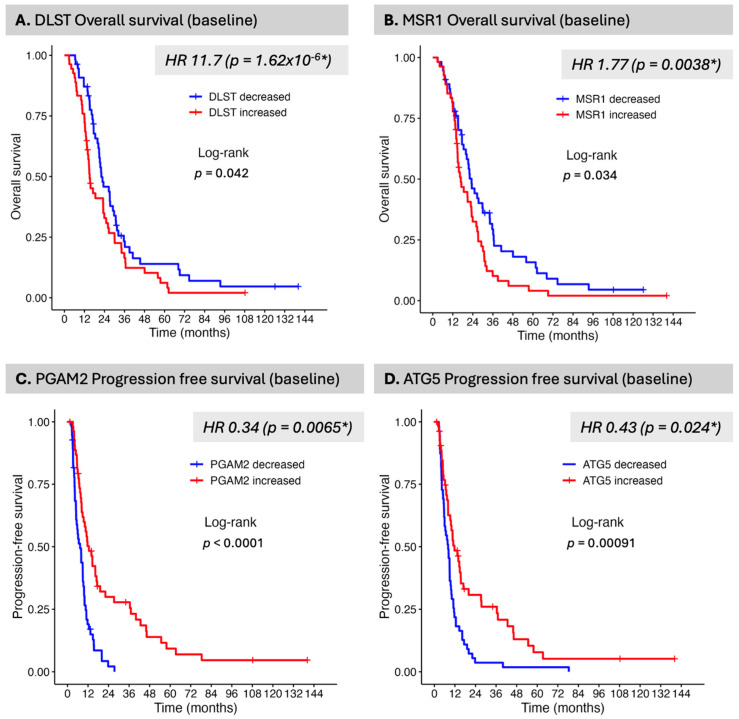
Selected proteins associated with overall survival (OS) or progression-free survival (PFS) adjusted by age, VPA (Valpoic Acid), and GTVT1 (gross tumor volume on T1 Gadolinium-enhanced MRI sequence). Univariate Cox proportional hazards models were fit to test individual protein expression pre-treatment levels for association with both OS and PFS. (**A**) DLST (dihydrolipoamide S-succinyltransferase), a component of the pyruvate dehydrogenase complex, was the only protein associated with OS after adjustment for multiple comparisons (FDR *p* < 0.05). (**B**) MSR1 (Macrophage Scavenger Receptor-A, aka CD204), emerged in baseline OS, PFS, and in its alteration with OS in the regression analysis but did not meet FDR threshold for significance. It was associated with OS in the Kaplan–Meier analysis. PGAM2 (Phosphoglycerate Mutase 2) (**C**) andATG5 (autophagy-related gene 5) (**D**) represent examples of two proteins that are highly associated with PFS, with similar directionality despite not meeting FDR threshold in the regression analysis. Both are associated with p21 kinase. * indicates statistical significance.

To verify whether the noted behavior of DLST in association with survival was maintained, a separate Kaplan–Meier survival analysis was performed for DLST as well as several additional proteins identified in Table 1, Supplementary File S1, particularly focusing on proteins with pivotal pathway assignment in relationship to outcome. A baseline (pre-CRT) lower DLST level was associated with an improvement in OS, consistent with the finding in the previous Cox analysis (*p* = 0.042) (Figure 1A). Additional significant proteins were also identified in the Kaplan–Meier analysis. A lower MSR1 was associated with an improvement in OS (*p* = 0.034) (Figure 1B). In the initial survival analysis, MSR1 was statistically significantly associated with OS, with an HR of 1.8 and a *p*-value of 0.004; however, the adjusted *p*-value was high (0.75). With respect to PFS, the identified proteins all had high false discovery rates (Supplementary File S1) but in the Kaplan–Meier survival analysis several proteins were associated with statistically significant improvement in PFS. In the Kaplan–Meier analysis, we noted that some proteins were significant irrespective of percentile cut-offs while others were only significant at select cut-offs or not at all. We focused our attention on proteins whose pre-CRT level was significant across percentile ranges (select proteins visualized in Supplementary Figure S3). PGAM2 (Phosphoglycerate Mutase 2) and ATG5 (autophagy-related gene 5) represent two such proteins in contrast to AKT1 and MGMT (Supplementary Figure S3). PGAM2 and ATG5 (Figure 1C,D) both had higher levels similarly associated with improvement in PFS. SPHK1 was, similarly, also statistically significant; however, the significance was not reflected in the Kaplan–Meier analysis (Supplementary Figure S4).

### 3.3. Metabolomic Data Analyses

In the metabolomic data set, only 318 (5.3%) out of 6015 compounds had a level 1 annotation, indicating the highest level of certainty for biological annotation based on the identification of peaks in the SECIM database. Of these, only 205 (3.4%) also had an assigned KEGG ID. The remainder of the compounds were either annotated with biological annotation at level 3 certainty (there is no level 2 annotation) based on HMDB metid or were identified by a mass-to-charge ratio and retention time in the data output. All compounds were initially included in the analysis; however, the focus of the analysis was on level 1 compounds, given their known biological annotation.

Analysis of the metabolomic data revealed several hundred compounds associated with the outcome (Supplementary Figure S1 and Supplementary File S2), with several specific compounds emerging as significant, as indicated by adjusted *p*-values, including 7-HOCA (Table 2). An elevated 7-HOCA level at baseline was associated with detriment in both PFS (HR 1.5) and OS (HR 1.3). P-cresol was the top identified pre- vs. post-CRT altered compound; however it did not meet the FDR significance threshold (Table 2). An elevated p-cresol pre- versus post-CRT was associated with HR less than 1, indicating a possible improvement in both PFS (HR 0.9) and OS (HR 0.8). Comparing pre- vs. post-CRT levels, 7-HOCA did not emerge significantly (Table 2, Supplementary File S2). Baseline (pre-CRT) levels S1P (Sphingosine 1-phosphate), meanwhile, were associated with both PFS and OS, and for alteration with PFS (Table 2); however, the adjusted *p*-value did not meet significance. In the Kaplan–Meier survival analysis, a decreased S1P level was associated with improvement in PFS (Supplementary Figure S4).

**Figure 2:**
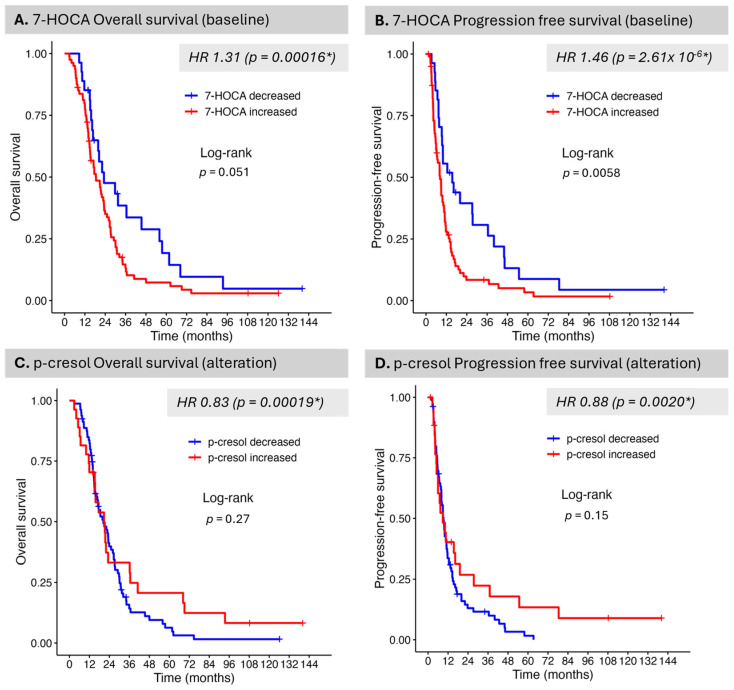
Selected compounds associated with overall survival (OS) or progression-free survival (PFS) adjusted by age, VPA, and GTVT1. Univariate Cox proportional hazards models were fit to test individual compound expression pre-treatment levels (**A**,**B**) and alteration pre- vs. post-CRT (**C**,**D**) for association with OS (**A**,**C**) and PFS (**B**,**D**). Pre-CRT, 7-HOCA (7-alpha-hydroxy-3-oxo-4-cholestenoate), was the top statistically significant compound for both OS (**A**) and PFS (**B**). 7-HOCA is a bile acid and cholesterol metabolite and putative GB biomarker. P-cresol alteration pre- vs. post-CRT was the top statistically significant compound for both OS (**C**) and PFS (**D**). p-cresol has been identified as a potential biomarker in cerebrospinal fluid (CSF). Outcome from Cox regression adjusted by age, VPA, and GTV T1. Overall survival and progression-free survival for baseline 7-HOCA (**A**,**B**). Overall survival and progression-free survival for alterations in p-cresol (**C**,**D**). * indicates statistical significance.

In the Kaplan–Meier survival analysis, baseline 7-HOCA and alteration in p-cresol were both associated with outcome, with decreased baseline serum levels of 7-HOCA being beneficial to both OS and PFS (25% percentile levels shown); however, statistical significance was observed only for PFS (*p* = 0.0058) (Figure 2A,B). An increased p-cresol level showed a trend toward improvement in OS and PFS in Kaplan–Meier analysis (75% percentile levels shown); however, this did not meet statistical significance (Figure 2C,D).

### 3.4. Pathway Analysis

**Figure 3:**
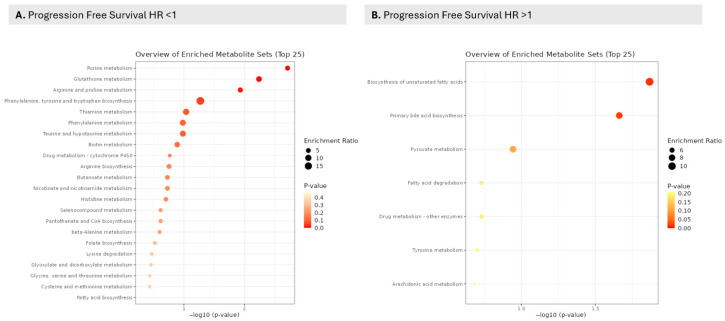
Overview of metabolite-set enrichment (top 25 pathways). Dot plot summarizing over-representation analysis of the metabolomics dataset. The x-axis shows −log10(*p*-value); dot size reflects the enrichment ratio (observed/expected hits per set), and color denotes significance (warmer/red = smaller P). Labels list the top enriched sets; only the 25 most significant are displayed. The enrichment overview of metabolic pathways associated with the serum metabolomic signal and progression-free survival was based on MSEA analysis of metabolites with available KEGG IDs. Panel (**A**) illustrates pathways for progression free survival (PFS) with HR < 1. An enrichment in purine metabolism is associated with decreased likelyhood of progression. Conversely panel (**B**) illustrates enrichment observed that is associated with a detriment in PFS (HR > 1), with biosynthesis of fatty acids associated with an increased likelyhood of progression.

**Figure 4:**
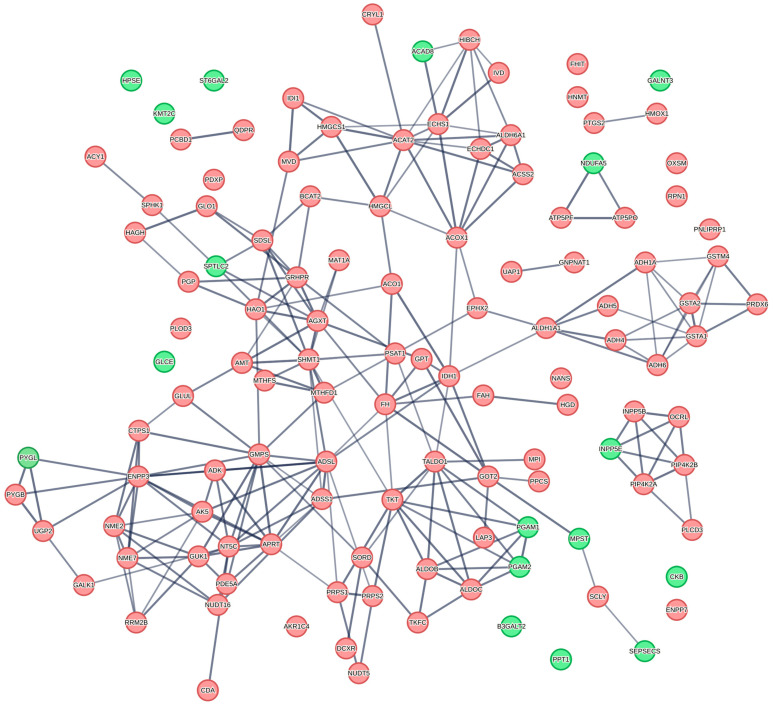
Protein–protein interaction network (STRING) of proteins from metabolically enriched pathways associated with progression-free survival. Proteins with HR > 1 are colored in red, while proteins with HR < 1 are colored in green. Most proteins associated with metabolic pathways show hazard ratios (HR) greater than 1, suggesting that elevated pre-CRT proteomic expression in metabolic pathways is linked to poorer progression-free survival outcomes. Nodes represent proteins; edges indicate STRING functional associations, with line thickness reflecting interaction confidence. Clustering highlights metabolic modules (e.g., carbohydrate and amino acid metabolism). Network generated at string.org; labels show the top connecting proteins and modules relevant to the PFS signal [31].

Given the need to both verify relationships between proteins and probe for a deeper understanding of biological pathway relationships which may be employed for downstream targeting, pathway analysis for proteins and metabolites associated with OS and PFS was carried out. Several metabolic pathways were identified in connection with PFS and OS (Table 3). PFS was highly statistically significantly associated with fatty acid, amino acid and purine metabolism as well as pathways centered on carbohydrate metabolism. At the same time, OS metabolic pathways were connected to phenylalanine, ethanol, fructose, and mannose metabolism, with fatty acid metabolism not being statistically significant (Table 3). Both PFS and OS outcome endpoints were associated with amino acid metabolism across several metabolic pathways. In relation to PFS, purine, glutathione, arginine, and proline metabolism had the lowest FDRs for HR < 1, and biosynthesis of fatty acids and primary bile acid biosynthesis were identified for HR > 1; however, FDRs were very high at 90% (Table 4, Figure 3, Supplementary File S3). Overall, purine metabolism was associated with an improvement in PFS, while fatty acid metabolism was associated with a detriment in PFS. The proteins related to metabolic pathways in the PFS analysis include several proteins implicated in the purine metabolic pathway (Figure 4), including those associated with poor PFS (Table 5). Using the metabolome data, MSEA analysis for pathways associated with survival revealed high false discovery rates for OS with spermidine and spermine biosynthesis and metabolism of amino acids and derivatives, sharing the lowest FDR at 20% (Supplementary File S3). Relating identified pathways to proteins responsible for the pathway hits (Table 5) revealed several critical mediators using STRING visualization of relationships between fatty acid metabolism (upper nodes, Figure 4, central node ACAT2) and purine metabolism (lower left corner nodes, Figure 4, ADSL) (Table 5).

### 3.5. MGMT and IDH Class Comparisons

**Figure 5:**
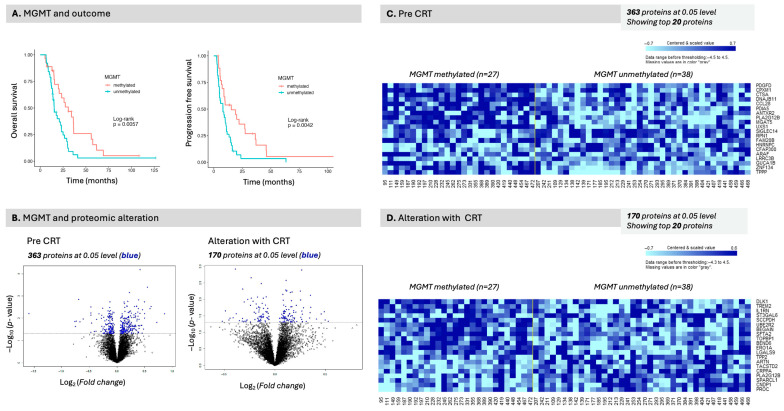
MGMT status class comparison of differentially expressed proteins. (**A**) MGMT status and outcome, overall survival (left panel) and progression-free survival (right panel). MGMT status was statistically significant for both outcome measures. (**B**) Proteomic differences were observed between classes both pre-CRT (363 proteins) and in alteration pre- vs. post-CRT (170 proteins). Pre-CRT signal is illustrated in (**C**) and alteration with CRT in (**D**).

MGMT methylation status was known in 60% of patients and IDH mutation status was known in 38%. MGMT methylation status was associated with outcome, both OS (*p* = 0.0057) and PFS (*p* = 0.0042) (Figure 5A). While several proteins were identified in the MGMT class comparison (Figure 5B), the FDRs, however, were high (Figure 5B). The top identified protein was PLAG12B (Phospholipase A2 Group XIIB) (*p* < 0.001, FDR 0.47) which can be seen in the heatmaps in Figure 5C,D and all the identified proteins are supplied in Supplementary File S3.

**Figure 6:**
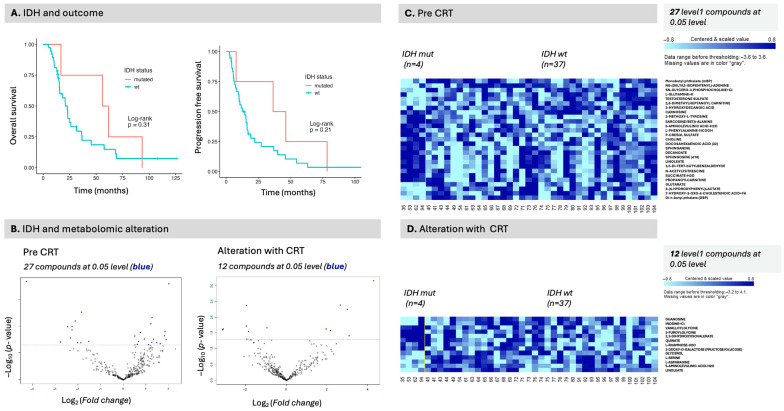
IDH status class comparison of differentially expressed compounds. (**A**) IDH status and outcome, overall survival (left panel) and progression-free survival (right panel). IDH status was not statistically significant for either outcome measure, although exhibiting the expected trend. Only four patients were IDH-mutated. (**B**) Metabolomic differences were observed between classes both pre-CRT (27 proteins) and in alteration pre- vs. post-CRT (12 proteins). Pre-CRT signal is illustrated in (**C**) and alteration with CRT in (**D**).

In the pathway analysis, the class comparison for MGMT status revealed a significant association with metabolic pathways, specifically amino acid and purine metabolism, as well as fatty acid degradation (Table 6, Supplementary File S4). By contrast, relatively few compounds were identified as compared to the number of differentially expressed protein signals (Supplementary Figure S5). In relationship to outcomes, IDH-mutated patients (*n* = 4) performed better than IDH wild-type patients; however, this difference was not statistically significant due to the small sample size (Figure 6A). In the IDH class comparison, several hundred compounds were identified, including several level 1 compounds; however, fewer proteins (Figure 6B, Supplementary Figure S6). The identified level 1 compounds are visualized in the heatmaps Figure 6A (pre) and Figure 6B (alteration).

**Figure 7:**
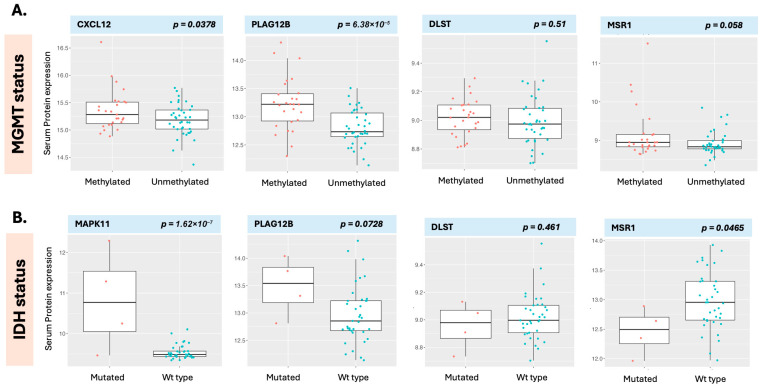
Proteins are differentially expressed in the MGMT class comparison (**A**) and the IDH class comparison (**B**). PLAG12B (second panel from the left) was statistically significantly differentially expressed in relationship to both MGMT and IDH with MGMT-methylated and, interestingly, IDH-mutated individuals both exhibiting higher levels of the molecule. DLST and MSR1 (right hand panels) were both associated with overall survival in earlier analyses but only MSR1 exhibited borderline statistical significance for IDH status.

The class comparison analysis enabled the comparison of proteins and compounds that were differentially expressed in the MGMT (Figure 7A) and IDH (Figure 7B) class comparison analyses. While a multitude of proteins were differentially expressed between classes (Supplementary File S4), we chose several significant proteins as well as proteins identified in the survival analysis, DLST and MSR1 (Figure 7, right panels) for comparison of measured levels between molecular classification classes, MGMT and IDH. PLAG12B (Phospholipase A2 Group XIIB) was statistically significantly differentially expressed based on molecular classification, with MGMT methylation associated with elevated expression in MGMT-methylated patients compared to unmethylated patients (*p* < 0.0001) (Figure 7A). Similarly, CXCL12 was also increased in patients with methylated tumors (*p* = 0.04) (Figure 7A). While PLAG12B was elevated in both MGMT-mutated and IDH-mutated patients exhibiting similar directionality, the difference between classes was not statistically significant in the IDH-mutated patients (Figure 7B). CXCL12, meanwhile, was not associated with IDH status; however, other critical proteins such as MAPK11, were. This illustrates different proteomic signal behavior between classes. Equally so, proteins associated with survival such as DLST levels, which were statistically significant for OS in the earlier analysis, were not statistically different in either the MGMT or IDH classes. MSR1, also associated with OS, was overall higher in methylated patients but did not meet statistical significance (*p* = 0.058) (Figure 7A).

In contrast, it was lower in IDH-mutated patients, which did meet statistical significance (*p* = 0.0465) (Figure 7B). Given that only four patients were IDH-mutated, this interpretation is less robust. In a separate analysis, aiming to examine the behavior of these molecules in an independent data set, we compared MSR1 expression levels with those from CPTAC (Clinical Proteomic Tumor Analysis Consortium) (tissue proteome), TCGA (The Cancer Genome Atlas Program) (genomic expression), and serum in healthy individuals (data provided by Somalogic). In the CPTAC data, we found that decreased MSR1 resulted in a superior outcome, similar to our serum-based data, while this was not observed in the TCGA data (Supplementary Figure S7). When comparing serum data from patients with GB with that were from healthy individuals, MSR1 was not statistically different in the GB serum data compared to healthy individuals. At the same time, DLST, SPHK1, MGMT, IDH, and AKT1 were all elevated in patients with GB as compared to healthy individuals (Supplementary Figure S8).

**Figure 8:**
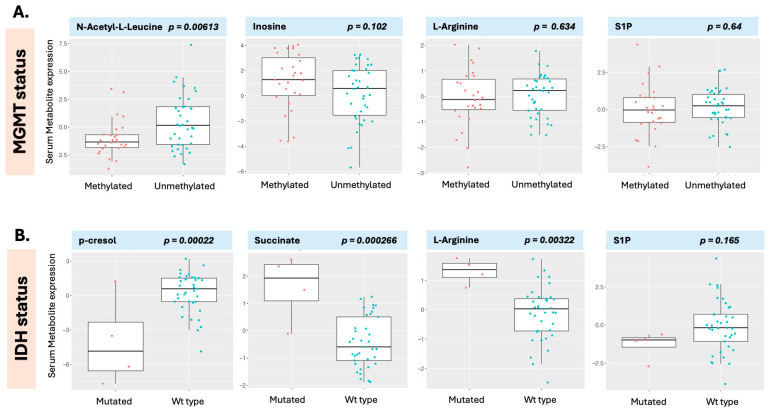
Metabolites were differentially expressed in the MGMT class comparison (**A**) and the IDH class comparison (**B**). N-acetyl-L-Leucine was decreased in patients with MGMT-methylated disease while p-cresol was decreased in patients with IDH-mutated disease. S1P (sphingosine-1-phosphate), was statistically significant for both overall and progression-free survival (Supplementary Figure S3); however, it was not associated with either MGMT or IDH status. L-arginine was increased in patients with IDH-mutated disease but exhibited no relationship to MGMT status.

For IDH status, several metabolic pathways were identified in relation to the proteomic signal (Table 7). Several metabolites were significantly altered in IDH-mutated patients, including p-cresol and 7-HOCA (both seen in Figure 6C, compound labels, right panel) and p-cresol in Figure 8B, left hand panel, as well as several proteins (Supplementary Figure S7). Unmethylated patients had higher serum N-acetyl-L-leucine levels (Figure 8A), whereas IDH wild-type patients had higher serum p-cresol levels but lower levels of succinate and L-arginine (Figure 8B), illustrating differential expression of metabolites between molecular classes. S1P levels, which were found significant for both survival and progression in the earlier analysis (Supplementary Figure S4), were not significantly different between classifications for MGMT or IDH (Figure 8, right panels), once more indicating that proteins associated with survival may not be associated with molecular classification or conversely that markers associated with molecular classification may not exhibit a direct relationship to outcome in serum.

## 4. Discussion

The present analysis involves serum biospecimens collected before and after completion of CRT in patients with pathologically proven GB. Four patients in this cohort were subsequently found to be IDH-mutated and would now be classified as astrocytoma IDH-mutated. The cohort spans 20 years during which first MGMT and then IDH became recognized molecular classifiers that were translated into the clinic, leading to data acquisition in some but not all patients, based on when they were diagnosed. We evaluated progression and overall survival, comparing MGMT-methylated patients with unmethylated patients, as well as IDH-mutated patients with IDH wild-type patients.

**Figure 9:**
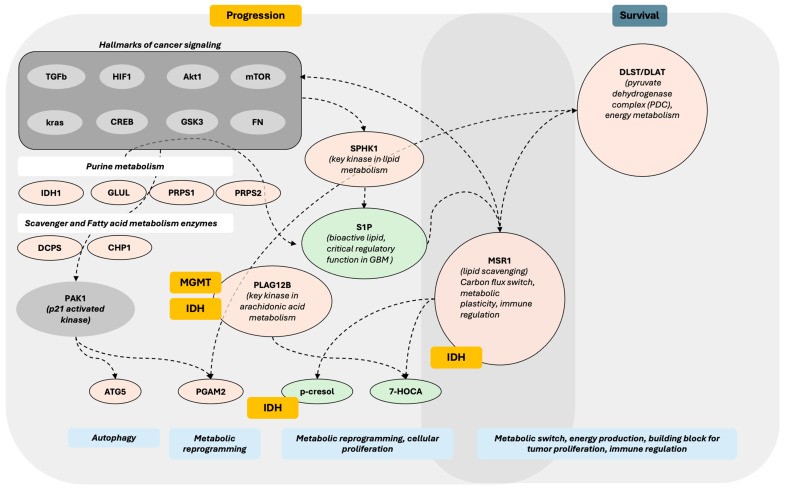
Postulated signaling pathway based on serum-identified proteomic and metabolomic signals in GB. Proteins (tan) and compounds (green) identified in the analysis are shown in relationship to each other. Hallmarks of cancer signaling proteins with their known literature connections are illustrated in grey boxes. Proteins and compounds associated with molecular classifications are marked with orange boxes, MGMT and or IDH based on the results of the present analysis. The left aspect of the figure illustrates signals associated with progression, and the right aspect is of association with survival (DLST (dihydrolipoamide S-succinyltransferase), a component of the pyruvate dehydrogenase complex), while MSR1 (Macrophage Scavenger Receptor-A, aka CD204) straddles both outcome measures.

DLST was the only serum protein associated with overall survival to meet the significance threshold after adjustment for multiple comparisons. DLST (dihydrolipoamide S-succinyltransferase) is a component of the pyruvate dehydrogenase complex. In the present analysis, an elevated baseline serum DLST level was associated with decreased survival and was the only protein with a favorable FDR to do so. DLST is a mitochondrial critical component of the pyruvate dehydrogenase complex with significant implications for glucose metabolism (Figure 9). We were unable to validate this finding using CPTAC tissue proteome data or TCGA genomic data. DLST was, however, significantly elevated in the serum of patients with GB compared with healthy individuals. Similarly, an increased MSR1 level emerged in baseline OS, PFS, and in its alteration with OS in the regression analysis and although it did not meet FDR threshold for significance, it was associated with OS in the survival analysis. MSR1 is an interesting molecule as we also noted that it has similar directionality in the CPTAC tissue proteome data, but not in the TCGA genomic data. MSR1 was, however, not significantly different in the serum of patients with GB as compared with healthy individuals. MSR1 (Macrophage Scavenger Receptor-A, aka CD204), has been found elevated in GB as compared to other cancers; its expression is associated with glioma progression [32], and increased levels at the tissue level have been associated with poorer survival outcomes in several cancers, and the serum data in the present analysis support this. MSR1 is a membrane glycoprotein with complex functionality that intersects between lipid metabolism and immune regulation (Figure 9) [32,33]. has been identified as an independent prognostic factor in TCGA and CGGA, and is associated with immune-associated gene sets [33]. MSR1 is DLST and MSR1 emerged as prominent molecules in the present analysis but indicate that validation with independent datasets will be protein-dependent. A protein may be behaving similarly in serum and in tissue in relationship to the disease states (MSR1), while another may be providing specificity with altered detection in a disease state, as in this case of GB vs. healthy individuals (possible example DLST). Additional proteins of interest identified in the present analysis include PGAM and ATG5. While neither of these had a significant FDR in the initial analysis, both had pre-CRT levels that were significant for PFS, in contrast to other more prominent molecules such as MGMT and AKT1. This finding indicates that it is critical to examine signals by multiple different means (regression and survival analysis) to enhance detection of potentially hidden biomarkers. PGAM2 (Phosphoglycerate Mutase 2), for example converts 3-phosphoglycerate to 2-phosphoglycerate in the glycolytic pathway, a critical step that mediates glycolysis, the primary mode of energy generation in malignant conditions. PGAM2 is phosphorylated by PAK1, marking it for ubiquitin-mediated degradation [34]. PGAM2 is upregulated in several cancers and can be critically increased by an alternative glycolytic pathway originating via a cancer-specific isoform of pyruvate kinase [35]. PGAM2 activity is also linked to metabolic programming, including the regulation of phospholipid metabolism [35]. Meanwhile, ATG5 (autophagy-related gene 5) is a proapoptotic molecule [36] that has been demonstrated to be linked to the immune system and increased in the presence of hypoxia in GB and astrocytoma. It has also been linked to protective autophagy, a hypoxia-induced phenomenon in glioma. Studies have shown that knockdown of ATG5 decreases cell mobility and increases chemosensitivity in hypoxic conditions via the HIF1α/ATG5 axis [37] and the CREB/ATG5 axis [38]. ATG5 has been detected in serum and proposed as an early biomarker of malignant mesothelioma [36], with an elevated level associated with activated autophagy. This process may occur via the acetylation of PAK1 (p21-activated kinase 1), which leads to autophagy and tumor proliferation by phosphorylating ATG5, thereby protecting ATG5 from ubiquitination-dependent degradation [39]. In the present study, elevated serum ATG5 and PGAM2 were both highly statistically significantly associated with improved progression-free survival, which indicates a possible role for these molecules as potential biomarkers with avenues for validation given that they both have a relationship to p21-activated kinase 1. PAK1 is not currently present in the 7K Somalogic panel and therefore could not be directly measured but with growing proteomic datasets, this may change. It is possible that ATG5 degradation was more prevalent in patients with superior tumor biology, leading to its detection in serum and observed improved outcome. It is also possible that both signals originate from a cellular population that is either less hypoxic or less driven by protective autophagy, or both, hence benefiting from an improved outcome independent of treatment. Elevated levels of serum PGAM2 and ATG5 were associated with improved progression-free survival in a similar manner. Since both are directly linked to p21-activated kinase 1, which is heavily implicated in cancer, this also leads to potential regulation with GSK3 and Akt1 [40] and ubiquitination-dependent degradation, again potentially providing upstream avenues for validation. This process may, presumably, increase the ability for detection in serum, as observed in the present study. PAK1 is a p21-activated kinase associated with GB development, hypoxic conditions, and poorer survival, characterized by increased invasiveness, cellular proliferation, and autophagy [39]. The postulated interaction (Figure 9) links PAK1 and, via phosphorylation, ATG5 and PGAM2, subsequently driving autophagy and glycolysis, respectively (Figure 9).

Despite a relatively much smaller set of measured metabolites that are biologically annotated and KEGG-linked, several were identified as critical in connection with OS, PFS, and IDH mutation status. 7-alpha-hydroxy-3-oxo-4-cholestenoate (7-HOCA) is a bile acid intermediate and cholesterol metabolite implicated in the classical pathway of bile acid synthesis occurring in the liver. Recent evidence has shown that 7-HOCA may represent a promising biomarker in GB. It was detected in both CSF and plasma with 7-HOCA levels found elevated in both biospecimen type, in patients with GB as compared to healthy individuals. 7-HOCA levels were associated with an increased risk of GB [41]. This finding is supported by serum data in the present study with increased levels associated with detriment in OS and PFS. Previous research has also shown that 7-HOCA can be employed as a marker of blood–brain barrier dysfunction, with data supporting the flux of 7-HOCA from the brain against a concentration gradient, given its preferential binding to albumin [42]. 7-HOCA is converted to CDCA (chenodeoxycholic acid) and cholic acid, metabolites that were also identified as associated with PFS and OS in the present analysis. The observed hazard ratios were greater than 1 for these metabolites, similar to 7-HOCA, before CRT and less than 1 after CRT, indicating that CRT administration impacts the bile acid signaling pathway and its various enzymatic steps, resulting in an improvement in outcome when these metabolites are decreased. The present study revealed primary bile acid biosynthesis, the biosynthesis of unsaturated fatty acids, and arachidonic acid metabolism across several analyses based on metabolomic serum signals. From a proteomic signal standpoint, PLA2G12B, a critical enzyme in arachidonic acid metabolism, was differentially expressed between MGMT-methylated patients, with increased levels observed in these patients. This finding links arachidonic acid metabolism to both outcome and molecular classification in GB. The brain is highly dependent on cholesterol for its function, accounting for approximately 20% of the body’s total cholesterol, and uniquely synthesizes cholesterol de novo. Under conditions of hypoxia, enzymatic activity leads to the biosynthesis of fatty acids and cholesterol [41].

We identified serum p-cresol as being associated with outcome, as well as IDH status, in the present study. P-Cresol has also been previously identified as a potential marker of IDH status in CSF [43]. The class comparison analysis for IDH status, while exploratory, nonetheless provides valuable insights into differences based on IDH status. Similar to the work of Möhn et al., p-cresol levels were decreased in patients with an IDH mutation compared to patients with an IDH wild-type, providing the literature validation. We note that IDH status is not present in many large glioma databases with the exception of TCGA [44] and also not present in either RTOG0525 [45] or RTOG0825 [46]. When present, a relatively small number of patients are IDH-mutated [47], which can render data analysis in this space and in serum particularly valuable. In upstream signaling (Figure 9), S1P (sphingosine-1-phosphate) has been implicated in several cancers, including GB [48]. In the present study, it has been associated with PFS, as has SPHK1 (Sphingosine Kinase 1), which is responsible for rendering S1P a bioactive lipid. Elevations in S1P and SPHK1 were both associated with a higher risk of progression in the analysis; however, this association was not statistically significant in the Kaplan–Meier survival analysis. SPHK1 and S1P provide an instrumental connection to lipid metabolism, as well as MSR1, the metabolites p-cresol and 7-HOCA, and a means of validation in other biospecimens and datasets.

These multi-omic analyses indicate that CRT produces measurable, clinically relevant shifts in GB biology, with GB linked biomarkers tracking patient outcome. Although only one pre-CRT protein, DLST, remained significant for OS after multiple-testing correction (higher baseline DLST predicting markedly worse OS), convergent Kaplan–Meier findings reinforce a treatment effect: lower baseline DLST and MSR1 aligned with improved OS, and higher baseline PGAM2 and ATG5 associated with longer PFS. The metabolomics further support CRT impact on systemic metabolism. P-cresol was the top pre- vs. post-CRT altered compound and showed a benefit (HR < 1 for both OS and PFS), while decreased baseline 7-HOCA favored better PFS (and trended for OS). S1P also tracked with outcome (lower baseline levels associating with improved PFS), and several analytes exhibited percentile dependent significance, consistent with heterogeneous therapy responses. Together, these patterns suggest that CRT not only selects prognostic molecular states present at baseline possibly via purine and fatty acid metabolism but also drives post-treatment metabolic reprogramming detectable in circulation which may at least partly be mediated by lipid metabolism. The present analysis builds further rationale for dynamic monitoring of serum samples in larger cohorts of patients.

To translate the findings from this study, we propose a clinical validation pathway integrating multi-omic, imaging, and longitudinal sampling. Initial steps include analytical validation of a serum assay panel (p-cresol, S1P, 7-HOCA, DLST, MSR1, PGAM2, and ATG5) as well as known hallmarks of cancer proteins with standardized pre-analytics and central IDH and MGMT testing. We also propose parallel analysis of IDH status for all patients where tissue is available and blood collection at multiple timepoints on existing trials. A prospective observational study would aim to collect serum at multiple timepoints (pre-CRT, during, post-CRT, and follow-up) and fuse metabolite trajectories with MRI radiomics to track therapy response and progression. Parallel mechanistic studies can correlate serum S1P and SPHK1 activity with tumor tissue and CSF to confirm biological linkage. Subsequent biomarker-guided adaptive trials could test whether monitoring of metabolites, such as p-cresol, are feasible and can be utilized to alter management and outcomes. Addressing data gaps, particularly the limited IDH-mutant representation requires central genotyping, targeted accrual, and Bayesian modeling to ensure robust subgroup inference. To move proteomic and metabolic markers from retrospective discovery toward clinically actionable tools for dynamic glioma monitoring, integrating omics with imaging and molecular profiling is critical and should form a cornerstone of ongoing and upcoming trials in glioma.

Limitations of the current study include its retrospective nature and the period covered, which spans over 20 years during which diagnostic standards, including the prevalence of MGMT and IDH testing, have changed. MGMT and IDH status were missing in 40% and 59% of the cohort, potentially limiting conclusions that can be made without further validation. Only four patients were IDH-mutated, which may also limit the conclusions that can be drawn regarding the IDH class comparison.

## 5. Conclusions

This study identifies serum p-cresol and 7-HOCA as circulating metabolites associated with both clinical outcome and in the case of p-cresol, IDH status in glioma, corroborating prior cerebrospinal fluid findings and extending evidence to a minimally invasive biospecimen. In conjunction with lipid pathway markers such as S1P and SPHK1, and metabolic enzymes including DLST, MSR1, PGAM2, and ATG5, our results reveal that chemoradiation (CRT) induces measurable, systemic metabolic reprogramming linked to prognosis. These data highlight a coordinated shift in purine, fatty acid, and lipid metabolism that reflects tumor biology and treatment response. Despite the retrospective nature of the cohort and incomplete molecular annotation, particularly limited IDH-mutant representation, the intersection of multi-omic and outcome findings provides strong biological rationale for prospective validation. Future studies should integrate longitudinal serum sampling, metabolic imaging fusion, and multi-specimen analyses to refine these biomarkers into dynamic, clinically actionable tools. The proposed translational pathway can allow for future standardized assay development to adaptive, biomarker-guided trial design, which, in turn, can accelerate clinical adoption. Collectively, these findings position serum metabolomics, anchored by p-cresol and 7-HOCA and lipid metabolism intermediates, as a promising avenue for real-time disease monitoring in glioma.

## Figures and Tables

**Table 1:** Top 15 proteins with adjusted *p* value (employing adjustment for age, valproic acid (VPA), and gross tumor volume on T1 Gadolinium-enhanced MRI (GTV-T1)) associated with progression-free and overall survival in relationship to the baseline (pre-CRT) and with alteration pre- vs. post-CRT. Overall, only one protein (DLST, dihydrolipoamide S-succinyltransferase) met significance for OS after adjustment for multiple comparisons (FDR *p* < 0.05) (Supplementary File S1 shows all signals with associated unique sequence IDs for the measured proteins).

**Progression-Free Survival (Baseline)**				**Progression-Free Survival (Pre- vs. Post-CRT)**			
**Protein**	***p-Value***	**HR**	***p Adjusted***	**Protein**	***p-Value***	**HR**	***p Adjusted***
DCPS	1.3 × 10^−5^	4.9	7.5 × 10^−2^	ETNK1	1.2 × 10^−5^	24.7	8.4 × 10^−2^
DGCR2	2.1 × 10^−5^	0	7.5 × 10^−2^	LCK	5.3 × 10^−5^	11.2	1.8 × 10^−1^
ADH5	5.0 × 10^−5^	3.3	9.4 × 10^−2^	MAGEA6	8.9 × 10^−5^	0.1	1.8 × 10^−1^
CHP1	1.4 × 10^−4^	5.1	9.4 × 10^−2^	PLEKHO2	9.6 × 10^−5^	0.1	1.8 × 10^−1^
HSPA1A	9.0 × 10^−5^	2.4	9.4 × 10^−2^	ANGPTL1	2.3 × 10^−4^	3.6	2.3 × 10^−1^
KCTD2	1.1 × 10^−4^	2.1	9.4 × 10^−2^	CLUAP1	2.5 × 10^−4^	0.2	2.3 × 10^−1^
PPBP	1.3 × 10^−4^	0.2	9.4 × 10^−2^	HMGA2	1.7 × 10^−4^	7.6	2.3 × 10^−1^
RNASE13	6.4 × 10^−5^	2	9.4 × 10^−2^	RBM46	2.6 × 10^−4^	2.4	2.3 × 10^−1^
SDSL	1.4 × 10^−4^	4.5	9.4 × 10^−2^	TBK1	2.8 × 10^−4^	6.1	2.3 × 10^−1^
STXBP6	7.2 × 10^−5^	3.9	9.4 × 10^−2^	ADAMTS1	8.5 × 10^−4^	5	3.7 × 10^−1^
THOP1	1.3 × 10^−4^	2.5	9.4 × 10^−2^	AIF1	9.3 × 10^−4^	0.4	3.7 × 10^−1^
CHCHD10	2.3 × 10^−4^	1.7	1.1 × 10^−1^	CRISP3	8.2 × 10^−4^	7.3	3.7 × 10^−1^
DSCC1	2.8 × 10^−4^	2.2	1.1 × 10^−1^	DCN	9.3 × 10^−4^	3.4	3.7 × 10^−1^
ELL2	2.6 × 10^−4^	2.4	1.1 × 10^−1^	FCRL2	7.1 × 10^−4^	13.2	3.7 × 10^−1^
HSPA1A	2.5 × 10^−4^	2.6	1.1 × 10^−1^	IFNB1	5.6 × 10^−4^	16.3	3.7 × 10^−1^
**Overall Survival (Baseline)**				**Overall Survival (pre- vs. post-CRT)**			
**Protein**	***p-value***	**HR**	***p adjusted***	**Protein**	***p-value***	**HR**	***p adjusted***
DLST	1.6 × 10^−6^	11.7	1.2 × 10^−2^	IGFBP5	4.4 × 10^−5^	2	3.2 × 10^−1^
CYCS	3.8 × 10^−5^	4.2	1.3 × 10^−1^	ETNK1	1.8 × 10^−4^	39.1	3.7 × 10^−1^
ATL3	5.5 × 10^−5^	4.3	1.3 × 10^−1^	F5	2.3 × 10^−4^	1.8	3.7 × 10^−1^
IGFBP5	2.9 × 10^−4^	0.5	4.6 × 10^−1^	NPS	2.5 × 10^−4^	2.1	3.7 × 10^−1^
PFN4	3.1 × 10^−4^	4.8	4.6 × 10^−1^	PRSS8	2.5 × 10^−4^	2.9	3.7 × 10^−1^
OPHN1	4.1 × 10^−4^	1.7	4.9 × 10^−1^	SERPINF2	3.4 × 10^−4^	1.9	3.8 × 10^−1^
SERF1A	5.3 × 10^−4^	0.1	5.6 × 10^−1^	DCN	3.7 × 10^−4^	4	3.8 × 10^−1^
EXD1	6.7 × 10^−4^	2.3	5.6 × 10^−1^	SFRP5	4.3 × 10^−4^	1.5	3.9 × 10^−1^
IL1RAP	6.9 × 10^−4^	0.5	5.6 × 10^−1^	BAGE3	7.0 × 10^−4^	1.9	5.7 × 10^−1^
F5	8.4 × 10^−4^	0.6	6.1 × 10^−1^	GFRA1	1.1 × 10^−3^	1.8	7.2 × 10^−1^
CCN4	1.1 × 10^−3^	0.5	6.4 × 10^−1^	HEXB	1.2 × 10^−3^	1.8	7.2 × 10^−1^
HSD17B11	1.2 × 10^−3^	1.8	6.4 × 10^−1^	LST1	1.3 × 10^−3^	2.3	7.2 × 10^−1^
ABLIM3	1.3 × 10^−3^	1.8	6.4 × 10^−1^	TBK1	1.6 × 10^−3^	5.1	7.2 × 10^−1^
ADIPOQ	1.4 × 10^−3^	0.6	6.4 × 10^−1^	MAGEA6	1.6 × 10^−3^	0.1	7.2 × 10^−1^
AKT1	1.4 × 10^−3^	3.8	6.4 × 10^−1^	HMGA2	1.7 × 10^−3^	4.3	7.2 × 10^−1^

**Table 2:** Top 15 level 1 identified metabolites, with level 1 biological annotation, with adjusted *p* value (employing adjustment for age, valproic acid (VPA), and gross tumor volume on T1 Gadolinium enhanced MRI (GTV-T1)). The table indicates the compounds associated with progression-free and overall survival in relationship to the baseline (pre-CRT) and with alteration pre- vs. post-CRT. Baseline 7-HOCA (left table) and alteration in p-cresol (right table) were associated with outcome.

**Progression-Free Survival (Baseline)**				**Progression-Free Survival (Pre- vs. Post-CRT)**			
**Compound Name**	***p-value***	**HR**	***p adjusted***	**Compound Name**	***p-value***	**HR**	***p adjusted***
7-HYDROXY-3-OXO-4-CHOLESTENOIC ACID+FA	2.6 × 10^−6^	1.5	8.3 × 10^−4^	P-CRESOL SULFATE	2.0 × 10^−3^	0.9	2.4 × 10^−1^
ARACHIDONIC ACID (20)	1.2 × 10^−3^	1.3	1.2 × 10^−1^	PHENYLPYRUVATE+FA	2.4 × 10^−3^	1.3	2.4 × 10^−1^
GUANOSINE	1.7 × 10^−3^	0.9	1.2 × 10^−1^	CHENODEOXYCHOLATE-2H2O	3.0 × 10^−3^	0.9	2.4 × 10^−1^
Dipropylene glycol	1.7 × 10^−3^	1.2	1.2 × 10^−1^	3-AMINOSALICYLIC ACID	3.1 × 10^−3^	1.2	2.4 × 10^−1^
SPHINGOSINE-1-PHOSPHATE	1.9 × 10^−3^	1.3	1.2 × 10^−1^	HEPTADECANOATE+FA	6.4 × 10^−3^	1.5	4.1 × 10^−1^
CHENODEOXYCHOLIC ACID (CDCA)	2.6 × 10^−3^	1.2	1.2 × 10^−1^	SPHINGOSINE-1-PHOSPHATE	1.5 × 10^−2^	0.9	6.7 × 10^−1^
CHENODEOXYCHOLATE-2H2O	2.6 × 10^−3^	1.2	1.2 × 10^−1^	Dipropylene glycol	1.6 × 10^−2^	0.9	6.7 × 10^−1^
LYSO PC (16)+Na	3.8 × 10^−3^	1.3	1.5 × 10^−1^	L-METHIONINE SULFOXIMINE-NH3	1.7 × 10^−2^	0.9	6.7 × 10^−1^
MONO-(2-ETHYLHEXYL) PHTHALATE DIMER	8.1 × 10^−3^	1.2	2.3 × 10^−1^	CHENODEOXYCHOLIC ACID (CDCA)	1.9 × 10^−2^	0.9	6.9 × 10^−1^
INOSINE+Cl	9.3 × 10^−3^	0.9	2.3 × 10^−1^	2,6-DIMETHYLHEPTANOYL CARNITINE	2.5 × 10^−2^	0.9	7.8 × 10^−1^
ALDOPENTOSE	9.9 × 10^−3^	1.2	2.3 × 10^−1^	N-ACETYLGLYCINE +Na	2.8 × 10^−2^	1.2	7.8 × 10^−1^
P-CRESOL SULFATE	1.1 × 10^−2^	1.2	2.3 × 10^−1^	MALEIC ACID	3.5 × 10^−2^	1.1	7.8 × 10^−1^
LYSOPE (16)	1.1 × 10^−2^	1.2	2.3 × 10^−1^	LYSO PC (18)	4.0 × 10^−2^	0.9	7.8 × 10^−1^
LYSO PC (16)	1.1 × 10^−2^	1.2	2.3 × 10^−1^	ALDOPENTOSE	4.5 × 10^−2^	0.9	7.8 × 10^−1^
2-HYDROXYGLUTARATE	1.1 × 10^−2^	0.8	2.3 × 10^−1^	12(S)-HETE	5.0 × 10^−2^	0.9	7.8 × 10^−1^
**Overall Survival (Baseline)**				**Overall Survival (Pre- vs. Post-CRT)**			
**Compound Name**	***p-value***	**HR**	***p adjusted***	**Compound Name**	***p-value***	**HR**	***p adjusted***
7-HYDROXY-3-OXO-4-CHOLESTENOIC ACID+FA	1.6 × 10^−4^	1.3	5.0 × 10^−2^	P-CRESOL SULFATE	1.9 × 10^−4^	0.8	6.1 × 10^−2^
Dipropylene glycol	3.1 × 10^−4^	1.2	5.0 × 10^−2^	Dipropylene glycol	1.4 × 10^−3^	0.8	2.3 × 10^−1^
N-ACETYL-L-LEUCINE-H2O	1.9 × 10^−3^	1.2	2.0 × 10^−1^	CHENODEOXYCHOLATE-2H2O	2.2 × 10^−3^	0.9	2.4 × 10^−1^
RIBOFLAVIN	2.7 × 10^−3^	0.9	2.1 × 10^−1^	3-AMINOSALICYLIC ACID	6.1 × 10^−3^	1.2	3.3 × 10^−1^
ARACHIDONIC ACID (20)	9.3 × 10^−3^	1.2	5.0 × 10^−1^	2,6-DIMETHYLHEPTANOYL CARNITINE	6.2 × 10^−3^	0.8	3.3 × 10^−1^
DECANOATE	1.0 × 10^−2^	1.2	5.0 × 10^−1^	HEPTADECANOATE+FA	6.2 × 10^−3^	1.5	3.3 × 10^−1^
FORMYLKYNURENINE	1.1 × 10^−2^	1.3	5.0 × 10^−1^	Methylglutaryl-L-carnitine	1.7 × 10^−2^	0.9	5.8 × 10^−1^
ALPHA-AMINOADIPATE/N-METHYL-L-GLUTAMATE-NH3	1.6 × 10^−2^	1.1	6.2 × 10^−1^	ALPHA-AMINOADIPATE/N-METHYL-L-GLUTAMATE-NH3	1.7 × 10^−2^	0.9	5.8 × 10^−1^
PUTRESCINE-NH3	1.7 × 10^−2^	0.8	6.2 × 10^−1^	3-ALPHA,11-BETA,17-ALPHA,21-TETRAHYDROXY- 5-ALPHA-PREGNAN-20-ONE+FA	1.8 × 10^−2^	0.9	5.8 × 10^−1^
4-HYDROXYBENZENESULFONIC ACID	2.1 × 10^−2^	0.9	6.4 × 10^−1^	AZELAIC ACID-H2O	1.8 × 10^−2^	0.7	5.8 × 10^−1^
P-CRESOL SULFATE	2.4 × 10^−2^	1.1	6.4 × 10^−1^	4-PYRIDOXATE	2.1 × 10^−2^	1.1	6.1 × 10^−1^
SPHINGOSINE-1-PHOSPHATE	2.4 × 10^−2^	1.2	6.4 × 10^−1^	MEVALOLACTONE	2.7 × 10^−2^	1.1	6.1 × 10^−1^
METHYL BETA-D-GALACTOSIDE+Na	2.9 × 10^−2^	0.9	7.1 × 10^−1^	N-ACETYL-L-LEUCINE-H2O	2.8 × 10^−2^	0.9	6.1 × 10^−1^
L-HISTIDINE+Na	4.7 × 10^−2^	0.9	9.3 × 10^−1^	CHENODEOXYCHOLIC ACID (CDCA)	3.5 × 10^−2^	0.9	6.1 × 10^−1^
SEROTONIN-NH3	5.0 × 10^−2^	0.9	9.3 × 10^−1^	PANTOTHENIC ACID-H2O	3.6 × 10^−2^	0.9	6.1 × 10^−1^

**Table 3:** Serum proteome signals associated with pre-CRT and progression-free survival (PFS). The top 10 significantly enriched gene sets associated with PFS across 98 identified metabolic pathways are shown. Both PFS and OS outcome endpoints were associated with amino acid metabolism across several metabolic pathways. OS metabolic pathways were connected to phenylalanine, ethanol, fructose, and mannose metabolism, with fatty acid metabolism not statistically significant, while statistically significant for progression.

**PFS Top 10 Significantly Enriched Gene Sets in 98 Metabolic Pathways**		
**GeneList GeneSets**	**Number of Genes**	**KS Permutation *p*-Value**
Fatty_Acids_Oxidation,_mitochondrial	12	0.001
Glycolysis_and_Gluconeogenesis	41	0.002
Porphyrin_and_Heme_Metabolism	13	0.003
Pyruvate_Metabolism	6	0.007
Valine,_Leucine_and_Isoleucine_Metabolism	17	0.008
Xenobiotics_Metabolism	20	0.010
Purine_Biosynthesis	8	0.011
Alanine_and_Aspartate_Metabolism	12	0.042
AlanineandAspartateMetabolism	12	0.042
Glutamate_metabolism	15	0.049
**OS top 10 significantly enriched gene sets in 98 metabolic pathways**		
Fructose_and_Mannose_Metabolism	8	0.016
Phenylalanine_metabolism	10	0.002
Ethanol_Metabolism	10	0.011
Oxidative_Phosphorylation	16	0.054
Citric_Acid_Cycle	34	0.137
Fatty_Acids_Oxidation,_peroxisomal	13	0.176
Valine,_Leucine_and_Isoleucine_Metabolism	17	0.256
Methionine_Metabolism	13	0.291
Selenoamino_acid_metabolism	10	0.292
Folate_Metabolism	11	0.357

**Table 4:** Metabolic pathways associated with HR < 1 (less likely to progress) and HR > 1 (more likely to progress) based on over-representation analysis.

	Total	Expected	Hits	Raw *p*-Value	Holm *p*-Value	FDR
**PFS HR < 1**						
Purine metabolism	70.00	0.96	5	0.00194	0.155	0.155
Glutathione metabolism	28.00	0.38	3	0.00576	0.455	0.231
Arginine and proline metabolism	36.00	0.49	3	0.0117	0.913	0.312
**PFS HR > 1**						
Biosynthesis of unsaturated fatty acids	36.00	0.19	2	0.0136	1	0.873
Primary bile acid biosynthesis	46.00	0.24	2	0.0218	1	0.873

**Table 5:** Proteins responsible for linkages to the purine metabolic pathway, fatty acid metabolism, and primary bile acid biosynthesis. Several prominent purine pathway proteins and several fatty acid metabolism-related proteins as well as primary bile acid biosynthesis are associated with a HR > 1 indicating increased risk of progression.

Protein	*p*-Value	HR
**Purine metabolic pathway**		
ADSL	0.01	1.45
ADSS1	0.05	1.46
GLUL	0.01	1.46
IDH1	0.02	1.50
NUDT5	0.00	2.38
PRPS1	0.02	1.79
PRPS2	0.04	2.14
AK5	0.03	1.82
NME2	0.01	1.49
NT5C	0.04	2.06
**Fatty acid metabolism pathway**		
ACAT2	0.03	1.26
ACOX1	0.03	1.45
ADH1A	0.05	1.50
ADH4	0.01	1.32
ADH5	0.00	3.33
ADH6	0.00	1.96
ECHS1	0.01	1.31
**Primary bile acid biosynthesis**		
AKR1C4	0.05	1.19

**Table 6:** Serum proteome signals with differential expression and associated pathways based on MGMT-methylated vs. unmethylated status in GB.

Pathway Description	Number of Genes	KS Permutation *p*-Value
Metabolic pathways	576	0.00001
Valine, leucine, and isoleucine degradation	28	0.00008
beta-Alanine metabolism	16	0.02291
Pentose phosphate pathway	20	0.00309
Linoleic acid metabolism	15	0.05523
Primary bile acid biosynthesis	5	0.00128
Glycine, serine, and threonine metabolism	19	0.00463
Glycosaminoglycan degradation	16	0.00141
Fatty acid degradation	25	0.00066
Ether lipid metabolism	18	0.06592
Glyoxylate and dicarboxylate metabolism	11	0.00084

**Table 7:** Serum proteome signals with differential expression and associated statistically significant pathways based on IDH wild-type vs. mutated status in GB.

Pathway Description	Number of Genes	KS Permutation *p*-Value
Tryptophan metabolism	21	0.001
Glycosaminoglycan degradation	16	0.002
Fatty acid degradation	25	0.002
Propanoate metabolism	17	0.003
Tyrosine metabolism	31	0.003
Intestinal immune network for IgA production	40	0.004
Pentose and glucuronate interconversions	16	0.005
Viral myocarditis	32	0.007
Phototransduction	5	0.011
Glycolysis/Gluconeogenesis	57	0.011
Pyruvate metabolism	36	0.014
Arachidonic acid metabolism	30	0.016
Protein digestion and absorption	47	0.018
African trypanosomiasis	34	0.024
Graft-versus-host disease	34	0.026
Pancreatic secretion	59	0.030
Pentose phosphate pathway	20	0.037
Other glycan degradation	12	0.046

## Data Availability

The complete results are shared as part of the Supplementary Materials as additional files. The complete data will be shared once all analyses for outcomes are complete.

## Supplementary Materials

The following supporting information can be downloaded at https://www.mdpi.com/article/10.3390/curroncol32110650/s1; Supplementary Table S1. Patient characteristics (*n* = 109). Supplementary Figure S1. Forest plot for covariates for A. overall survival (OS) and B. progression-free survival (PFS) (109 patients). Supplementary Figure S2. Differentially expressed proteins and compounds adjusted by age, VPA, and GTV T1 in association with OS, PFS, MGMT status, and IDH status. Supplementary Figure S3. Progression-free survival analysis employing pre-CRT (baseline) serum levels of PGAM2 (A), ATG5 (B), MGMT (C), Akt1 (D) at the 25th, 50^th^, and 75th percentile. PGAM2 and ATG5 are significant in at least two (ATG5) or all (PGAM2) categories in contrast to MGMT and Akt1. Supplementary Figure S4. Overall and Progression-Free Survival Kaplan–Meier curves for the protein SPHK1 and the compound S1P, based on pre-CRT (baseline) data adjusted by age, VPA, and GTV T1. Supplementary Figure S5. Overall and Progression-Free Survival Kaplan–Meier curves for the proteins DLST and MSR1, based on serum pre-CRT (baseline) data (present analysis), the CPTAC tissue proteome data, and the TCGA dataset. Supplementary Figure S6. Histogram comparison of expression of critical proteins identified in the analysis in comparison with healthy individuals (SomaLogic provided healthy individuals’ aggregated dataset). Supplementary Figure S7. MGMT status class comparison of differentially expressed compounds. Supplementary Figure S8. IDH status class comparison of differentially expressed proteins. Supplementary files: Supplementary File S1. OS and PFS, pre- and post-alteration for proteome (4 tabs); Supplementary File S2. OS and PFS, pre- and alteration for metabolome (4 tabs); Supplementary File S3. MSEA pathways for OS and PFS using metabolome data and GSEA pathways for OS and PFS using the proteome data; Supplementary File S4. MGMT and IDH class comparisons (OS, PFS, pre- and alteration).

## Abbreviations

The following abbreviations are used in this manuscript:

CGGA	Chinese Glioma Genome Atlas
CPTAC	Clinical Proteomic Tumor Analysis Consortium
CRT	Concurrent chemoirradiation
CSF	Cerebrospinal fluid
GB	Glioblastoma
GTR	Gross Total Resection
GTV T1	Gross Tumor Volume on T1 Gadolinium-enhanced MRI sequence
IDH	Isocitrate Dehydrogenase
IMRT	Intensity Modulated Radiation Therapy
KPS	Karnofsky Performance Status
MGMT	O6-Methylguanine-DNA Methyltransferase
OS	Overall survival
PFS	Progression-Free Survival
RPA	Recursive Partitioning Analysis
RT	Radiation Therapy
STR	Subtotal Resection
TCGA	The Cancer Genome Atlas Program
TMZ	Temozolomide
VPA	Valproic Acid
