# Supplementary material for: Serum p-Cresol and 7-HOCA Levels and Fatty Acid and Purine Metabolism Are Associated with Survival, Progression, and Molecular Classification in GB—Serum Proteome and Metabolome Analysis Pre vs. Post Up-Front Chemoirradiation
Source: Curr Oncol. 2025 Nov 20;32(11):650. doi: 10.3390/curroncol32110650 (PMC12651722; doi:10.3390/curroncol32110650)
Supplement: Supplementary file 1 [file curroncol-32-00650-s001.zip › Supplemental Table 1.pdf]

Supplemental Table 1. Patient characteristics (*n=109*).

**Characteristics**		
Age	**Mean**	**Range**
	56.5	29-79
	**N = 109**	**%**
**Sex**		
Male	74	68%
Female	35	32%
**KPS**		
100	33	30%
90	46	42%
<80	23	21%
unknown	7	6%
**RPA**		
5	10	9%
4	77	71%
3	15	14%
unknown	7	6%
**Cortical/Periventricular**		
Cortical	66	61%
Periventricular	43	39%
**Type of Surgery**		
Biopsy only	9	8%
STR	62	57%
GTR	37	34%
Unknown	1	1%
**MGMT status**		
Methylated	27	25%
Unmethylated	38	35%
Unknown	44	40%
**IDH status**		
Mutated	4	4%
Wild type	37	34%
Unknown	68	62%
**Radiation technique**		
Rapid Arc	34	31%
IMRT	38	35%
3D conformal	36	33%
unknown	1	1%
**GTVT1 (cc)**		
	34.3	0-217
unknown	5	5%
**VPA**		
Yes	31	28%
No	78	72%
