# Supplementary material for: Serum p-Cresol and 7-HOCA Levels and Fatty Acid and Purine Metabolism Are Associated with Survival, Progression, and Molecular Classification in GB—Serum Proteome and Metabolome Analysis Pre vs. Post Up-Front Chemoirradiation
Source: Curr Oncol. 2025 Nov 20;32(11):650. doi: 10.3390/curroncol32110650 (PMC12651722; doi:10.3390/curroncol32110650)
Supplement: Supplementary file 1 [file curroncol-32-00650-s001.zip › Supplementary Figure 1.pptx]

## Slide 1
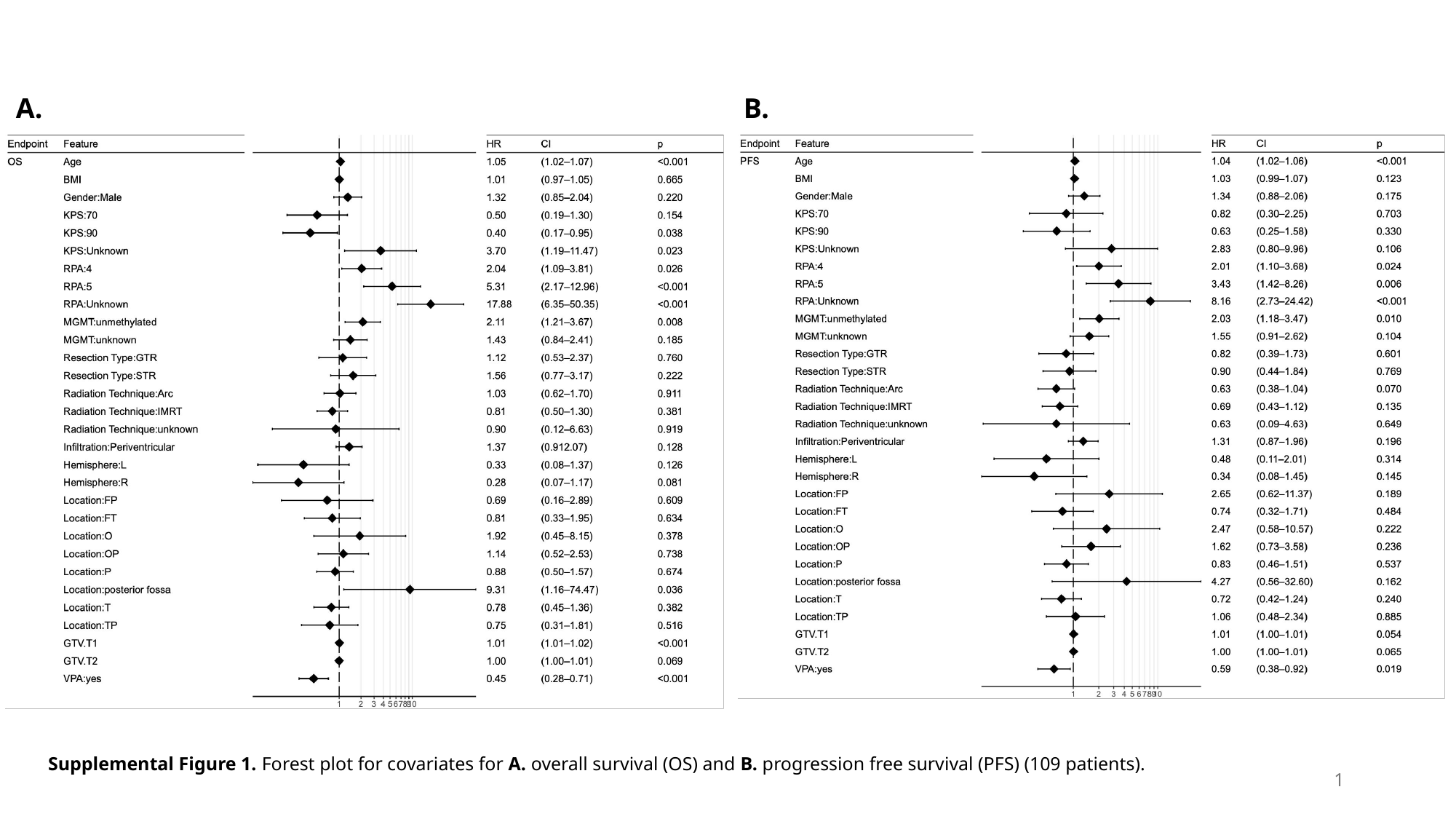

A.
B.
Supplemental Figure 1. Forest plot for covariates for A. overall survival (OS) and B. progression free survival (PFS) (109 patients).
1
