# Supplementary material for: Serum p-Cresol and 7-HOCA Levels and Fatty Acid and Purine Metabolism Are Associated with Survival, Progression, and Molecular Classification in GB—Serum Proteome and Metabolome Analysis Pre vs. Post Up-Front Chemoirradiation
Source: Curr Oncol. 2025 Nov 20;32(11):650. doi: 10.3390/curroncol32110650 (PMC12651722; doi:10.3390/curroncol32110650)
Supplement: Supplementary file 1 [file curroncol-32-00650-s001.zip › Supplementary Figure 2.pptx]

## Slide 1
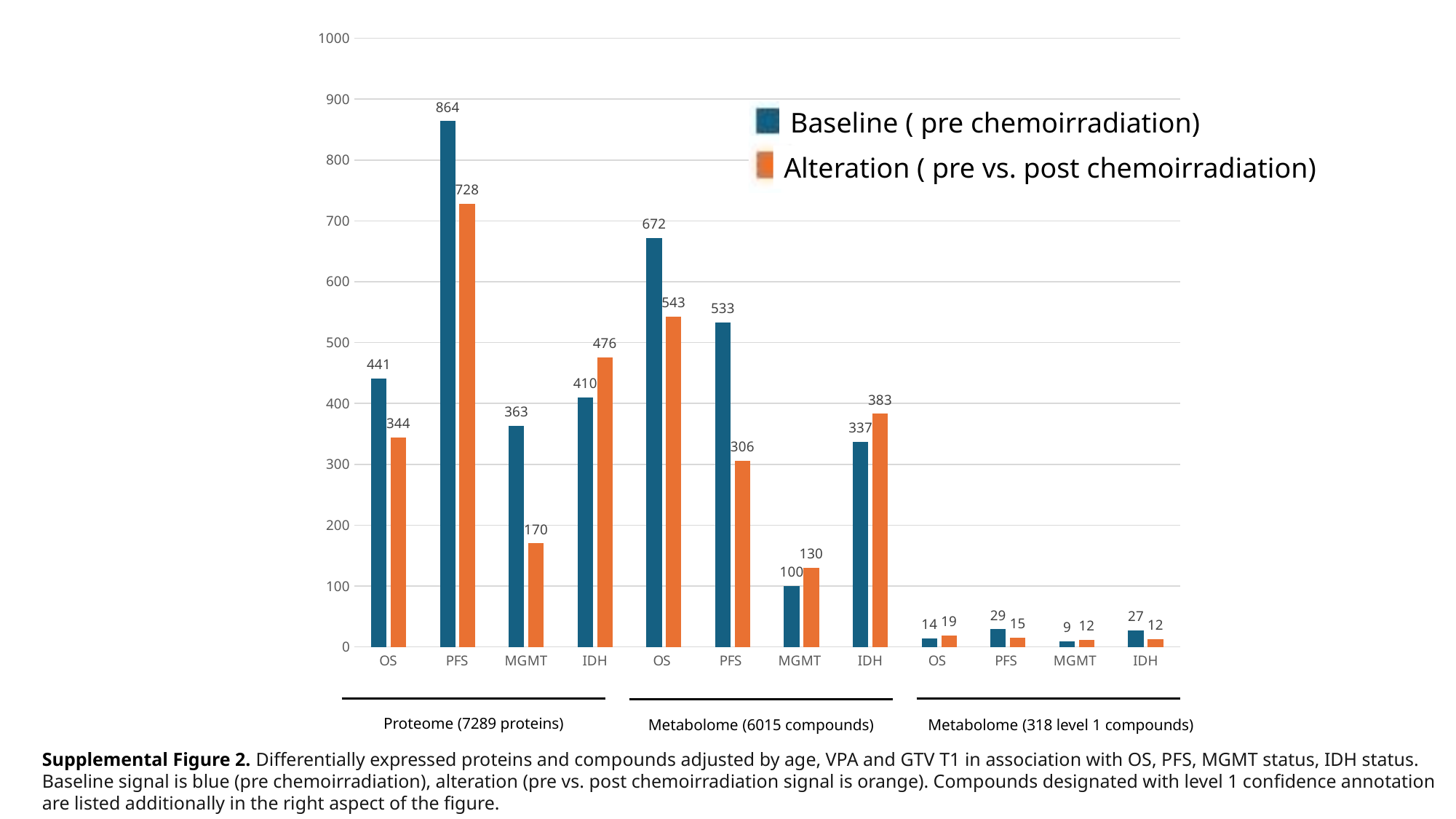

### Chart
| Category | Pre | Alt |
|---|---|---|
| OS | 441.0 | 344.0 |
| PFS | 864.0 | 728.0 |
| MGMT | 363.0 | 170.0 |
| IDH | 410.0 | 476.0 |
| OS | 672.0 | 543.0 |
| PFS | 533.0 | 306.0 |
| MGMT | 100.0 | 130.0 |
| IDH | 337.0 | 383.0 |
| OS | 14.0 | 19.0 |
| PFS | 29.0 | 15.0 |
| MGMT | 9.0 | 12.0 |
| IDH | 27.0 | 13.0 |
Baseline ( pre chemoirradiation)
Alteration ( pre vs. post chemoirradiation)
Proteome (7289 proteins)
Metabolome (6015 compounds)
Metabolome (318 level 1 compounds)
Supplemental Figure 2. Differentially expressed proteins and compounds adjusted by age, VPA and GTV T1 in association with OS, PFS, MGMT status, IDH status. Baseline signal is blue (pre chemoirradiation), alteration (pre vs. post chemoirradiation signal is orange). Compounds designated with level 1 confidence annotation are listed additionally in the right aspect of the figure.
1
