# Supplementary material for: Serum p-Cresol and 7-HOCA Levels and Fatty Acid and Purine Metabolism Are Associated with Survival, Progression, and Molecular Classification in GB—Serum Proteome and Metabolome Analysis Pre vs. Post Up-Front Chemoirradiation
Source: Curr Oncol. 2025 Nov 20;32(11):650. doi: 10.3390/curroncol32110650 (PMC12651722; doi:10.3390/curroncol32110650)
Supplement: Supplementary file 1 [file curroncol-32-00650-s001.zip › Supplementary Figure 3.pptx]

## Slide 1
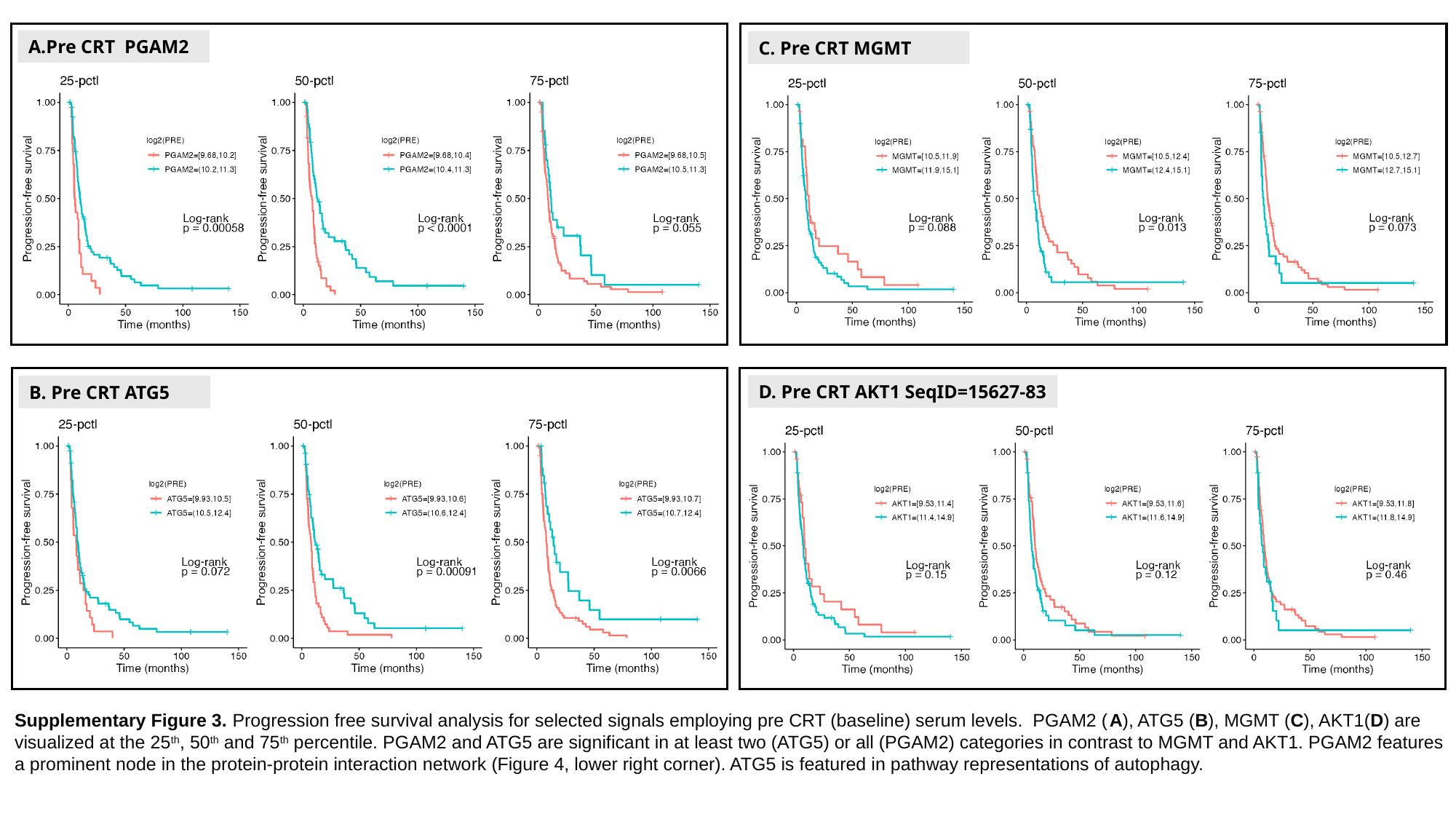

A.Pre CRT PGAM2
C. Pre CRT MGMT
D. Pre CRT AKT1 SeqID=15627-83
B. Pre CRT ATG5
Supplementary Figure 3. Progression free survival analysis for selected signals employing pre CRT (baseline) serum levels. PGAM2 (A), ATG5 (B), MGMT (C), AKT1(D) are visualized at the 25th, 50th and 75th percentile. PGAM2 and ATG5 are significant in at least two (ATG5) or all (PGAM2) categories in contrast to MGMT and AKT1. PGAM2 features a prominent node in the protein-protein interaction network (Figure 4, lower right corner). ATG5 is featured in pathway representations of autophagy.
