# Supplementary material for: Serum p-Cresol and 7-HOCA Levels and Fatty Acid and Purine Metabolism Are Associated with Survival, Progression, and Molecular Classification in GB—Serum Proteome and Metabolome Analysis Pre vs. Post Up-Front Chemoirradiation
Source: Curr Oncol. 2025 Nov 20;32(11):650. doi: 10.3390/curroncol32110650 (PMC12651722; doi:10.3390/curroncol32110650)
Supplement: Supplementary file 1 [file curroncol-32-00650-s001.zip › Supplementary Figure 5.pptx]

## Slide 1
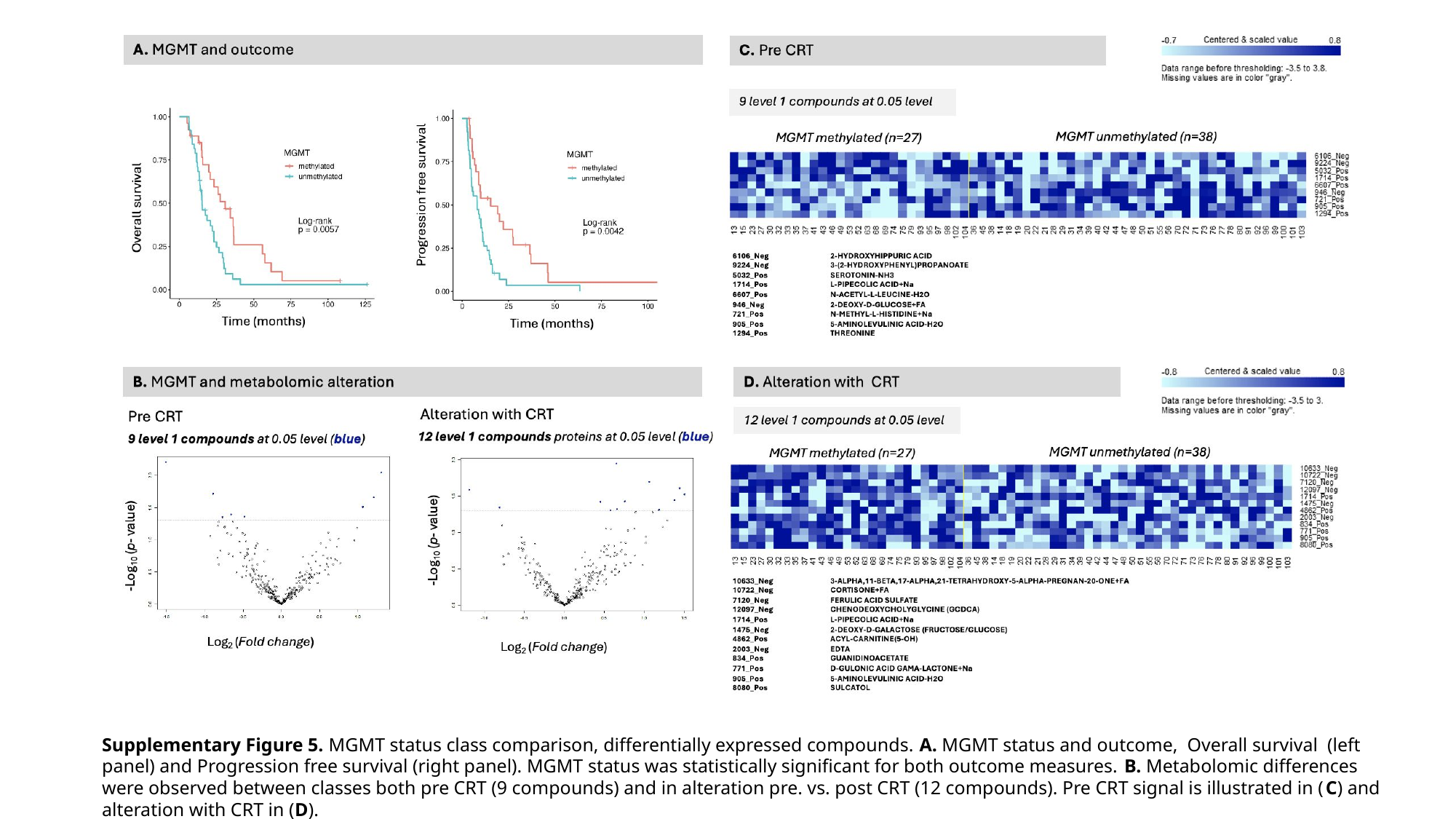

Supplementary Figure 5. MGMT status class comparison, differentially expressed compounds. A. MGMT status and outcome, Overall survival (left panel) and Progression free survival (right panel). MGMT status was statistically significant for both outcome measures. B. Metabolomic differences were observed between classes both pre CRT (9 compounds) and in alteration pre. vs. post CRT (12 compounds). Pre CRT signal is illustrated in (C) and alteration with CRT in (D).
