# Supplementary material for: Serum p-Cresol and 7-HOCA Levels and Fatty Acid and Purine Metabolism Are Associated with Survival, Progression, and Molecular Classification in GB—Serum Proteome and Metabolome Analysis Pre vs. Post Up-Front Chemoirradiation
Source: Curr Oncol. 2025 Nov 20;32(11):650. doi: 10.3390/curroncol32110650 (PMC12651722; doi:10.3390/curroncol32110650)
Supplement: Supplementary file 1 [file curroncol-32-00650-s001.zip › Supplementary Figure 6.pptx]

## Slide 1
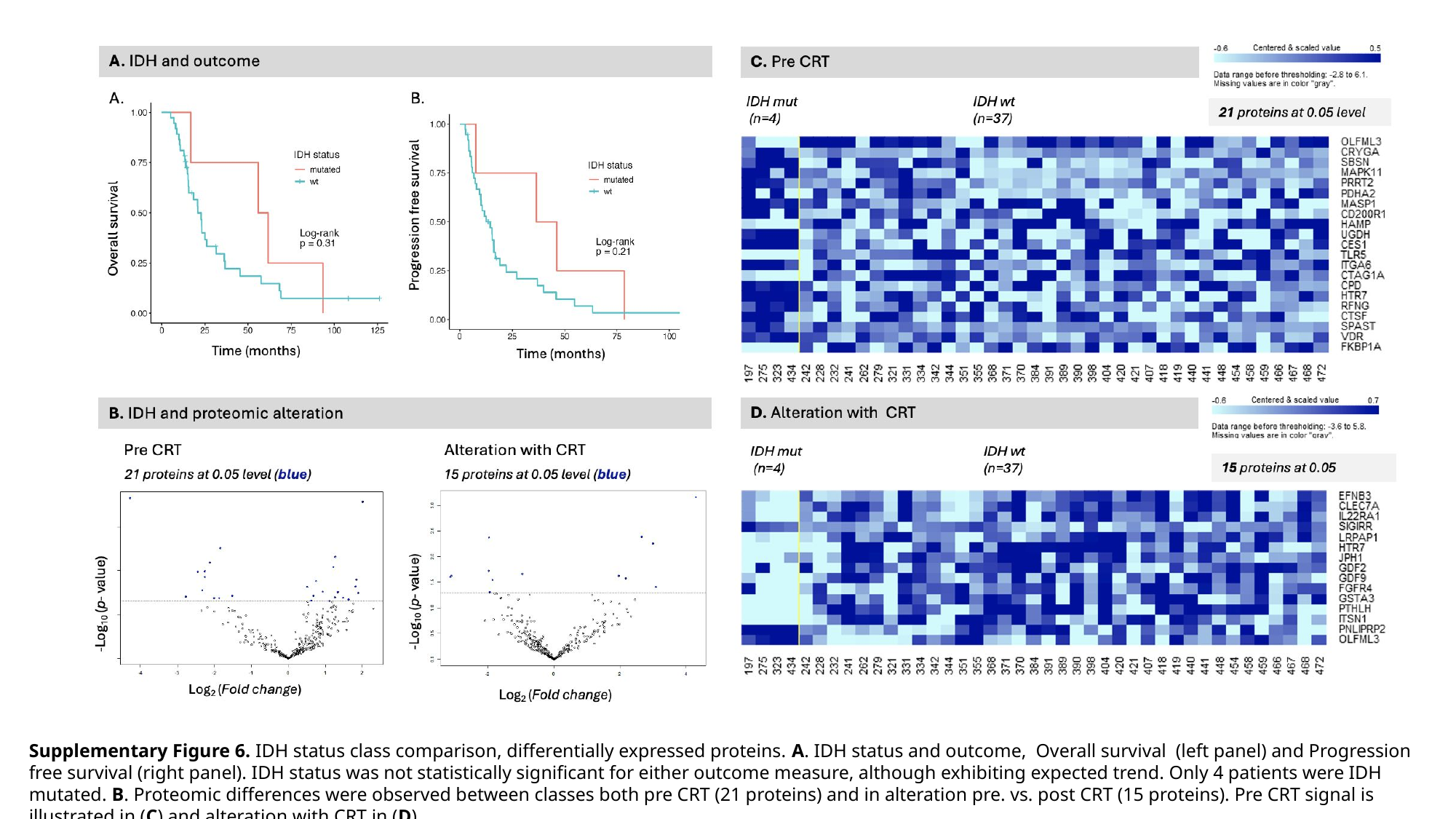

Supplementary Figure 6. IDH status class comparison, differentially expressed proteins. A. IDH status and outcome, Overall survival (left panel) and Progression free survival (right panel). IDH status was not statistically significant for either outcome measure, although exhibiting expected trend. Only 4 patients were IDH mutated. B. Proteomic differences were observed between classes both pre CRT (21 proteins) and in alteration pre. vs. post CRT (15 proteins). Pre CRT signal is illustrated in (C) and alteration with CRT in (D).
