# Supplementary material for: Serum p-Cresol and 7-HOCA Levels and Fatty Acid and Purine Metabolism Are Associated with Survival, Progression, and Molecular Classification in GB—Serum Proteome and Metabolome Analysis Pre vs. Post Up-Front Chemoirradiation
Source: Curr Oncol. 2025 Nov 20;32(11):650. doi: 10.3390/curroncol32110650 (PMC12651722; doi:10.3390/curroncol32110650)
Supplement: Supplementary file 1 [file curroncol-32-00650-s001.zip › Supplementary Figure 7.pptx]

## Slide 1
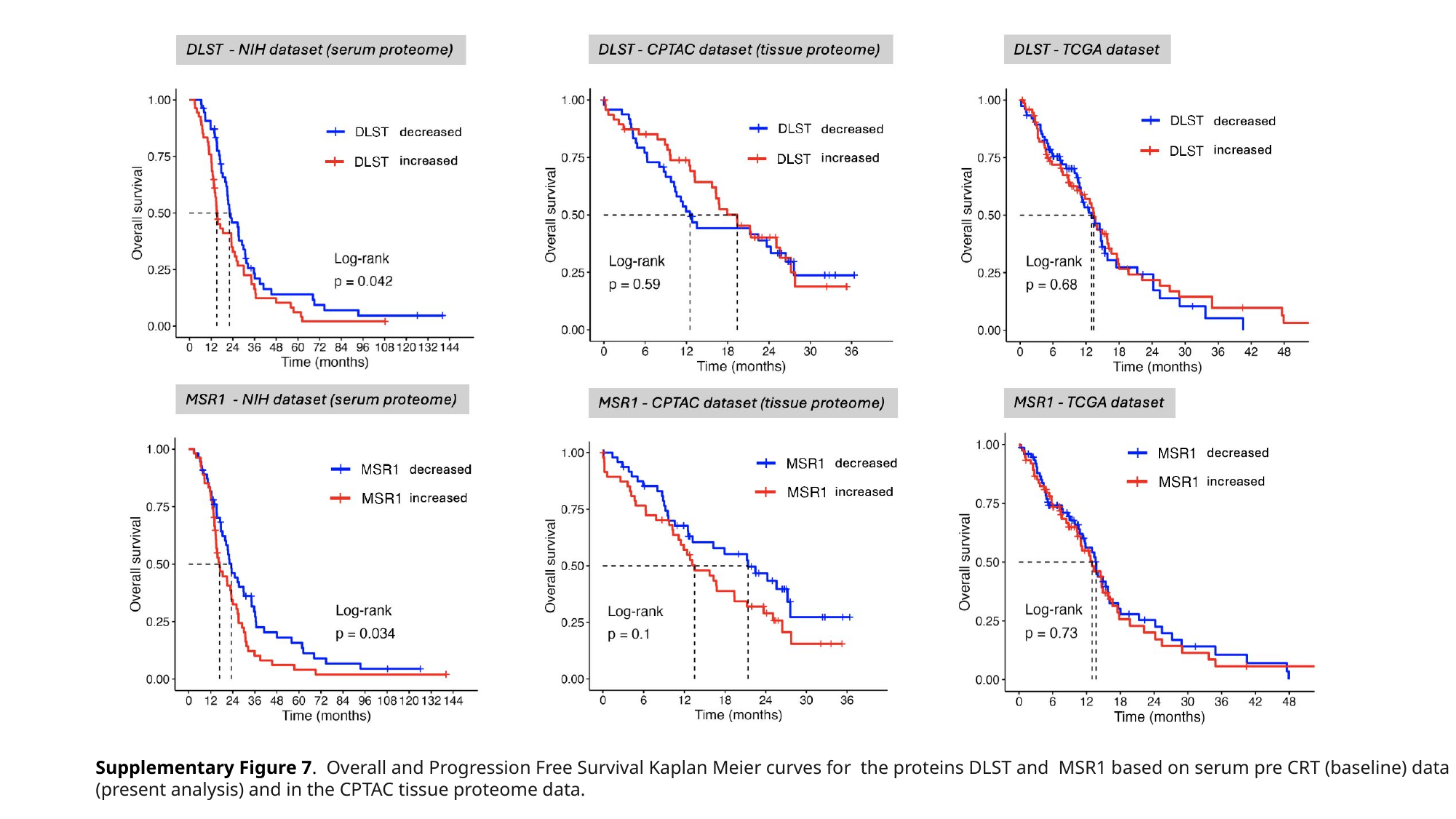

Supplementary Figure 7. Overall and Progression Free Survival Kaplan Meier curves for the proteins DLST and MSR1 based on serum pre CRT (baseline) data (present analysis) and in the CPTAC tissue proteome data.
